# Short-term effects of ocrelizumab on cortical pathology in patients with multiple sclerosis

**DOI:** 10.1016/j.neurot.2026.e00940

**Published:** 2026-06-08

**Authors:** N. Cavalli, G. Boffa, E. Cipriano, V.D. Boccia, S. Al Qudsi, F. Baldisseri, E. Leveraro, L. Nasone, S. Magon, M. Cellerino, M. Inglese, C. Lapucci

**Affiliations:** a Department of Neurosciences, Rehabilitation, Ophthalmology, Genetics and Maternal and Child Sciences, University of Genoa, Genoa, Italy; b IRCCS AOM San Martino Hospital, Genoa, Italy; c Department of Physics, University of Genoa, Genoa, Italy; d Pharma Research and Early Development, Neuroscience and Rare Diseases Roche Innovation Center Basel, F. Hoffmann-La Roche Ltd., Basel, Switzerland

**Keywords:** Multiple sclerosis, Ocrelizumab, Cortical lesions, AIDIR

## Abstract

Cortical lesions (CLs) are a common finding in multiple sclerosis (MS) but data regarding the impact of treatment on their evolution are lacking. This study evaluates CLs status and new CLs accrual over two-year follow-up in a cohort of patients treated with ocrelizumab (OCR) and the correlation with brain structural metrics and clinical measures. 87 relapsing-remitting MS patients [59 (67.8%) women, mean (SD) age 39.2 (10) years, median (IQR) baseline EDSS 2.5 (1.75)] underwent clinical, neuropsychological assessment and brain 3T MRI at baseline and two years after OCR start. CLs were manually segmented using Artificial Intelligence-Driven Imaging Reconstruction (AIDIR) sequences. At baseline, 21 patients (24.1%) had no CLs, 30 (34.5%) had 1 CL, 17 (19.5%) had 2 CLs, and 19 (21.8%) had ≥ 3 CLs. Higher CLs number and volume were associated with lower normalized TBV (r = −0.29, p = 0.01; r = −0.23, p = 0.03) and SDMT-raw score (r = −0.28, p = 0.02; r = −0.26, p = 0.03). After multiple-comparison correction, CLs number remained associated with normalized TBV. 14 patients (16.1%) experienced white matter lesions (WMLs) accrual and 15 patients (17.2%) disability progression over follow-up, but none of them developed new CLs. We observed the formation of only one CL at follow-up in a female patient that showed improved physical disability and no cognitive decline without concurrent WMLs accrual. OCR might also prevent lesion-driven cortical atrophy. These data seem to confirm the role of cortical pathology as an early marker of disease severity in MS, support OCR efficacy in minimizing lesion accrual and suggest distinct mechanisms underlying CLs and WMLs development.

## Introduction

Multiple sclerosis (MS) is the most common demyelinating disease of the central nervous system (CNS) and represents one of the leading causes of disability in young adults [1]. Once neglected due to poor detection with conventional neuropathological and neuroradiological methods [2], cortical lesions (CLs) have been rediscovered through immunohistochemistry and advanced MRI sequences [[3], [4], [5], [6]], which revealed them to be a consistent and widespread feature of MS, occurring from the earliest clinical stages and becoming prominent in progressive forms [[7], [8], [9]]. Their formal inclusion in the 2017 diagnostic criteria [10] now enables earlier and more accurate [4,11] diagnosis and distinction from other CNS disorders where CLs are scarce, such as NMOSD, MOGAD or migraine [[12], [13], [14]].

The presence of CLs further correlates with neurological disability [15,16], as measured by the Expanded Disability Status Scale (EDSS) [17]; in addition, it predicts the risk of clinical worsening [9] and is associated with poor cognitive performance [15,18]. CLs are best visualized using MRI techniques such as PSIR or DIR, which are rarely included in clinical evaluations protocols. Their widespread use is not technically feasible and impacts on the cost and the duration of the MRI scan.

Cortical pathology does not manifest only in the form of demyelinating lesions but also as atrophy, which is a well-known marker of physical disability and cognitive impairment [[19], [20], [21]]. It can be assessed by quantifying cortical volume or by measuring cortical thickness^22^.

The efficacy of disease-modifying therapies (DMTs) in reducing relapse frequency, decreasing new demyelinating lesions and limiting disability progression is well established [22,23]. Less well known, however, is the impact of DMTs on cortical pathology. Currently approved DMTs may reduce the occurrence of new CLs, although they do not seem to completely prevent neither their development, nor the accrual of cortical atrophy [[24], [25], [26]]. Nevertheless, data concerning the efficacy of more recent therapies on cortical pathology, including drugs that selectively deplete CD20^+^ B cells, are still limited.

Against this background, the aims of this study were: (a) to investigate the effect of ocrelizumab (OCR) on development of CLs and cortical atrophy in patients with relapsing-remitting (RR) MS; (b) to explore the relationship between CLs, brain structural metrics and clinical measures.

## Materials and methods

### Study population and clinical assessment

This is a single-center prospective longitudinal study including 87 consecutive patients with RR-MS who fulfilled the 2017 McDonald criteria [10] and initiated treatment with OCR between October 19, 2018 and December 30, 2021. All patients with MRI sequences of sufficient quality to allow AIDIR reconstruction and that underwent clinical, neuropsychological and neuroradiological assessments at baseline and at the two-year follow-up were included. Patients with contraindications to 3T MRI or to the use of contrast agents were excluded. OCR was prescribed and administered by the treating physician according to regulatory policies (https://www.gazzettaufficiale.it/atto/serie_generale/caricaDettaglioAtto/originario?atto.codiceRedazionale=25A00312&amp;atto.dataPubblicazioneGazzetta=2025-01-22&amp;elenco30giorni=false). All participants provided written informed consent and approval was obtained from the local ethical standards committee withing the framework of the Sys4MS project-ID43. We collected data from brain MRI, neurological evaluation and neuropsychological assessment both at the start of OCR therapy and after two years. Neurological disability was quantified by the EDSS [17], while cognitive impairment was evaluated by the Symbol Digit Modalities Test (SDMT). The number of correct responses within 90 s was recorded as the raw SDMT score (SDMT-r). Based on Italian normative data, raw scores were converted into t-scores and z-scores, standardized values adjusted for everyone’s age, sex and education. Patients with a baseline SDMT z-score below −1.5 were classified as impaired [27]. PIRA events were defined as disability progression independent of relapse activity, occurring when an increase in EDSS was observed either at least 90 days after a relapse or at least 30 days before a relapse [28]. The magnitude of EDSS increase required to define progression depended on the baseline score: an increase of at least 1.5 points was required for patients with a baseline EDSS of 0, an increase of at least 1.0 point for those with an EDSS between 1.0 and 5.0, and an increase of at least 0.5 points for patients with an EDSS ≥5.5. In all cases, progression had to be confirmed after at least 3 months from the visit documenting the initial EDSS increase [28]. No Evidence of Disease Activity 3 (NEDA-3) status was evaluated at follow-up.

### MRI protocol and processing

Subjects underwent 3T brain MRI (Siemens Prisma, Erlangen, Germany) with a 64-channel head and neck coil at baseline and two years after initiation of OCR. The MRI protocol at both timepoints included the following sequences:

1. 3D sagittal T2-fluid-attenuated inversion recovery (FLAIR) (TR/TI/TE 5000 ms/1800 ms/393 ms; original resolution: 0.8 × 0.8 × 1 mm^3^; reconstructed resolution: 0.4 × 0.4 × 1 mm^3^);
2. 3D sagittal T1-magnetization prepared rapid gradient-echo (T1-MPRAGE) (TR/TI/TE 2300 ms/919 ms/2.96 ms; resolution: 1 × 1 × 1 mm^3^);
3. 3D turbo spin-echo T1-weighted imaging following intravenous injection of a single dose of Gadoteridol (0.2 mL/kg) (TR/TE 700 ms/12 ms; resolution: 1 × 1 × 1 mm^3^).

CLs were manually segmented using Artificial Intelligence-generated Double Inversion Recovery (AIDIR) sequences based on high-resolution 3D-FLAIR and T1-MPRAGE [29,30]. The segmentation was performed by NC and reviewed by CL and GB. CLs were defined in accordance with the international consensus recommendations in this field as cortical hyperintensities which should occupy at least 3 pixels [31].

White matter lesions (WMLs) were segmented on FLAIR images using a semi-automated, user-supervised local thresholding approach (SinLab; Siena Imaging; https://sinlab-rhb.sienaimaging.com), and the results were visually reviewed by two experienced raters (NC and GB). Subsequently, 3D-FLAIR images were rigidly registered to T1-MPRAGE images with FSL (FMRIB Software Library, v6.0.5, Oxford, UK), and the resulting transformations were applied to align the FLAIR-hyperintense lesions to T1 space using nearest-neighbour interpolation. The T1-MPRAGE images were then lesion-filled using FSL. Using longitudinal FreeSurfer pipeline (version 7.4.1, https://surfer.nmr.mgh.harvard.edu/), we segmented the cortex into 68 cortical parcellations (34 for each hemisphere) based on the Desikan-Killiany atlas. At baseline, total brain volume (TBV) was calculated and normalized to the estimated total intracranial volume (eTIV). We quantified CL volume, cortical thickness and cortical volume of each cortical parcellation containing a cortical lesion.

### Statistical analysis

All statistical analyses were performed using Jamovi (2.3.28, https://www.jamovi.org). A p-value <0.05 was considered statistically significant. Descriptive statistics were reported as means with standard deviations (SD), medians with interquartile ranges (IQR) or number with percentage. Spearman correlation was used to investigate the association between the number of CLs and clinical, MRI and neuropsychological variables. The presence of new CLs and changes in CL load over follow-up were evaluated. Cortical volume loss and cortical thinning in regions containing CLs were analyzed; repeated measures ANOVA were performed focusing on regions where CLs were present, correcting only for the time interval between baseline and follow-up MRI scans.

## Results

### Demographic and clinical variables

Table 1 shows the demographic and clinical characteristics. The cohort included 87 patients, 67.8% female, with a mean age of 39.2 years (SD = 10) and a median EDSS of 2.5 (IQR = 1.75). MRI, clinical and neuropsychological assessments were performed at OCR initiation and again after two years of treatment. The mean time interval between baseline and follow-up MRI was 1.98 years (SD = 0.3). 15 (17.2%) patients experienced a PIRA event over follow-up. No Evidence of Disease Activity 3 (NEDA-3) was maintained by 60 (69%) patients.

**Table 1:** Demographic and clinical characteristics.

Data	Value
Number of patients	87
Sex M/F, number (%)	28/59 (32.2%/67.8%)
Age, years, mean (SD)	39.2 (10)
Baseline SMDT z-score, mean (SD)	0.47 (5.8)
Follow-up SDMT z-score, mean (SD)	0.1 (1.3)
Disease duration, years, mean (SD)	10.1 (10)
Baseline EDSS, median (IQR)	2.5 (1.75)
Follow-up EDSS, median (IQR)	2.5 (2)
Patients with ≥ 1 relapse in the year before OCR start, number (%)	46 (52.9%)
Patients with isolated MRI activity in the year before OCR start, number (%)	31 (35.6%)
Annualized relapse rate in the year before OCR start	0.53 (95% CI 0.38–0.68)
Naïve/previously treated, number (%)	24/63 (27.6%/72.4%)
Previous treatment with high efficacy/moderate efficacy, number (%)	46/17 (52.9%/19.5%)
Number of previous DMTs, median (SD)	1 (1.53)
Previous DMTs (%)	
- Fingolimod	− 21 (33.3)
- Alemtuzumab	− 9 (14.3)
- Natalizumab	− 8 (12.7)
- Cladribine	− 3 (4.8)
- Interferon	− 5 (7.9)
- Glatiramer	− 5 (7.9)
- Dimethyl fumarate	− 4 (6.4)
- Others	− 8 (12.7)

Note - M = male, F = female, SD = standard deviation, IQR = interquartile range, EDSS = Expanded Disability Status Scale, SMDT = Symbol Digit Modalities Test, OCR = ocrelizumab, DMTs = disease-modifying therapies.

### Cortical lesions assessment and association with disease severity

**Fig. 1:**
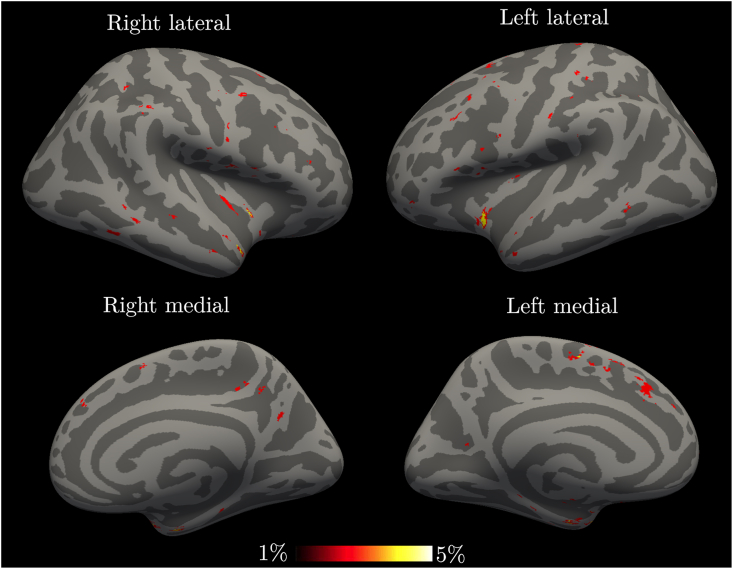
Cortical lesion probability map in patients with multiple sclerosis. Individual cortical lesion masks were projected onto the FreeSurfer fsaverage surface and combined to generate a vertex-wise lesion probability map, representing the frequency of lesion occurrence across patients.

MRI metrics are shown in Table 2. 21 (24.1%) patients had no CLs, 30 (34.5%) had 1 CL, 17 (19.5%) had 2 CLs, and 19 (21.8%) had ≥ 3 CLs. All CLs detected at baseline remained visible at follow-up. The lesion probability map showing the distribution of CLs is shown in Fig. 1. This map was obtained by projecting individual CLs masks onto the FreeSurfer *fsaverage* surface through surface-based registration and subsequently computing a lesion frequency map across patients. The majority of vertices affected by CLs were located, in descending order, in the following regions, according to the FreeSurfer Desikan–Killiany atlas: temporal pole, insula, superior frontal gyrus, parahippocampal gyrus, rostral middle frontal gyrus, caudal anterior cingulate cortex, precentral gyrus, precuneus, postcentral gyrus and paracentral lobule. The predominantly frontal and temporal distribution of CLs is consistent with previous findings reported in the literature [32]. Higher CLs number (Table 3) showed an association with lower normalized TBV (r = −0.29, p = 0.01) and lower SDMT-r (r = −0.28, p = 0.02); CLs number showed a trend toward association with SMDT z-score (r = −0.24, p = 0.05); no correlation was found between CLs number and disease duration (r = 0.14, p = 0.21). Higher total CLs volume at baseline (Table 4) was associated with lower normalized TBV (p = −0.23, p = 0.03) and lower SDMT-r (r = −0.26, p = 0.03); CLs volume at baseline showed a trend toward association with disease duration (r = 0.2, p = 0.06) and SDMT z-score (r = −0.23, p = 0.06). Neither baseline CLs number (r = 0.02, p = 0.83) nor CLs volume (r = 0.01, p = 0.93) showed an association with baseline EDSS. For the statistically significant correlations, we also applied correction for multiple comparisons using the Benjamini–Hochberg false discovery rate method. The correlation between CLs number and normalized TBV remained significant (r = −0.29, p = 0.02), while CLs number showed a trend toward association with SDMT-r (r = −0.28, p = 0.05). As for CLs volume, no correlations survived correction, although trends toward association were observed with normalized TBV (r = −0.23, p = 0.06) and SDMT-r (r = −0.26, p = 0.06).

**Table 2:** Baseline and follow-up MRI metrics.

Data	Baseline	Follow-up
Total number of CLs	169	170
Total number of leukocortical lesions	39	40
Total number of non leukocortical lesions	130	130
Mean number of CLs (SD) per patient	1.9 (2.5)	1.9 (2.5)
Mean number of leukocortical lesions (SD) per patient	0.4 (0.9)	0.5 (0.9)
Mean number of non leukocortical lesions (SD) per patient	1.5 (2)	1.5 (2)
Total CLs volume, ml	3.9	3.2
Total leukocortical lesions volume, ml	1.2	1
Total non leukocortical lesions volume, ml	2.7	2.2
Mean volume of CLs (SD) per patient, ml	0.1 (0.2)	0.1 (0.2)
Mean volume of WMLs (SD) per patient, ml	10.3 (10.2)	11 (10.8)

Note – WMLs = white matter lesions, CLs = cortical lesions, SD = standard deviation.

**Table 3:** Correlation between cortical lesion number and clinical/volumetric measures at baseline.

Cortical lesion number		
Variable	Correlation	p-value
Normalized TBV	−0.29	**0.01**
SDMT-raw	−0.28	**0.02**
SDMT z-score	−0.24	*0.05*
Disease duration	0.14	0.21
EDSS score	0.02	0.83

Note - SMDT = Symbol Digit Modalities Test, TBV = total brain volume, EDSS = Expanded Disability Status Scale.

**Table 4:** Correlation between cortical lesion volume and clinical/volumetric measures at baseline.

Cortical lesion volume		
Variable	Correlation	p-value
Normalized TBV	−0.23	**0.03**
SDMT-raw	−0.26	**0.03**
SDMT z-score	−0.23	0.06
Disease duration	0.2	0.06
EDSS score	0.01	0.93

Note - SMDT = Symbol Digit Modalities Test, TBV = total brain volume, EDSS = Expanded Disability Status Scale.

### Cortical lesion and white matter lesions assessment

**Fig. 2:**
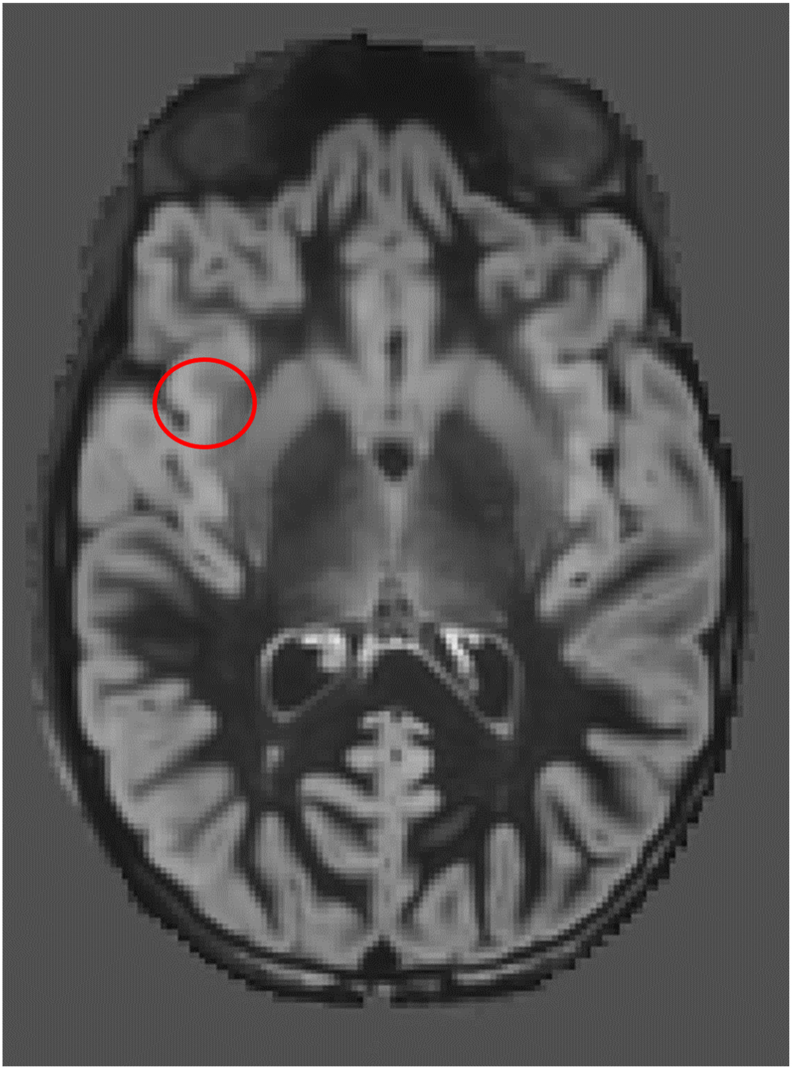
Selected axial AIDIR image from patient at baseline, the red circle highlights the region where a new cortical lesion developed at follow-up.

**Fig. 3:**
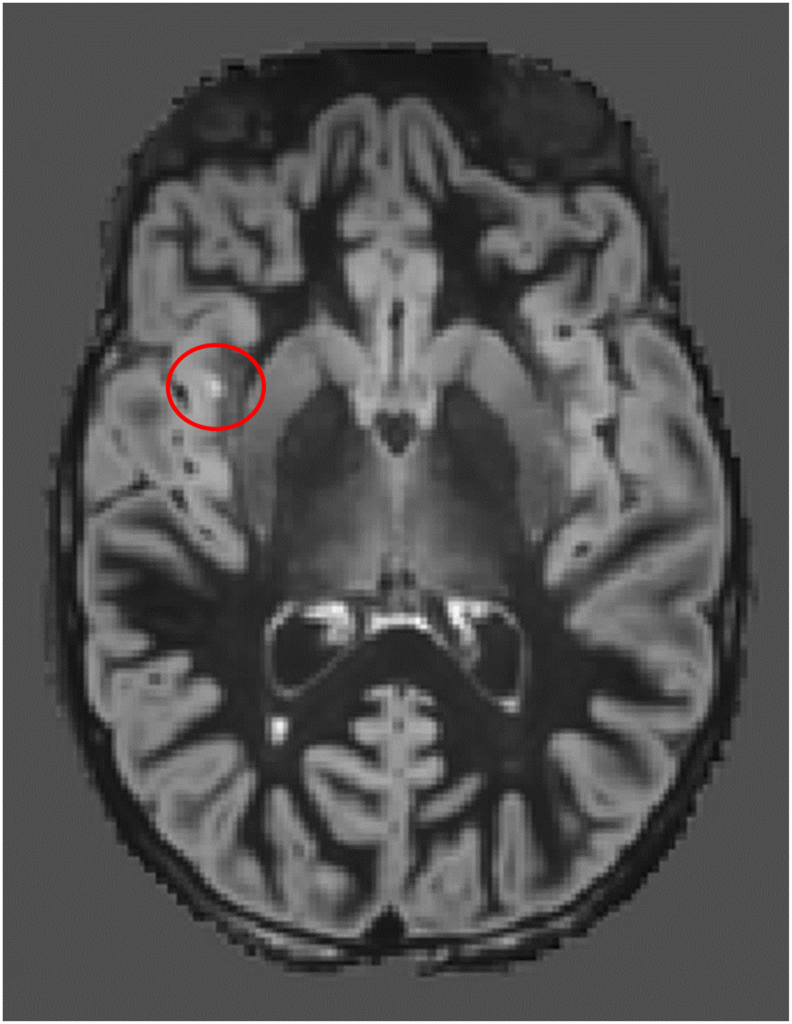
Selected axial AIDIR image from patient at follow-up, the red circle highlights the new cortical lesion.

3 patients (2.6%) presented a clinical relapse and 14 patients (16.1%) experienced WMLs accrual, mean (SD) per subject n = 1.4 (0.6) but none of them developed new CLs. Only one patient developed a single new CL during follow-up (Fig. 2, Fig. 3) without concurrent WMLs accrual. This was a 29-year-old female patient with disease duration of 2.9 years. She maintained NEDA-3 status, showed improvement in physical disability (EDSS 3.5 at baseline to 2.5 at follow-up) and no cognitive decline (z score −1.4 at baseline to −0.21 at follow-up).

### Locoregional atrophy analysis

Locoregional atrophy did not develop over two years. Focusing only on cortical regions with CLs, no statistically significant changes were observed in cortical volume (0.09 mL, p = 0.14, Table 5) or cortical thickness (0.002 mm, p = 0.9, Table 6) between baseline and follow-up. Patients without CLs at baseline (n = 21) and those with leukocortical lesions (n = 23) were excluded from this analysis.

**Table 5:** Cortical volume comparison between baseline and follow-up.

Cortical volume^a^		
Data	Value	p-value
Mean (SD) baseline, ml	9.15 (5)	0.14
Mean (SD) follow-up, ml	9.06 (4.89)	

Note - SD = standard deviation.

a Cortical volume of regions with cortical lesions.

**Table 6:** Cortical thickness comparison between baseline and follow-up.

Cortical thickness^a^		
Data	Value	p-value
Mean (SD) baseline, mm	2.61 (0.31)	0.9
Mean (SD) follow-up, mm	2.61 (0.26)	

Note - SD = standard deviation.

a Cortical thickness of regions with cortical lesions.

## Discussion

In this study, we confirmed that CLs are a common finding in patients with MS; specifically, 75.9% of our cohort showed the presence of at least one CL at baseline, values in line with those of other MRI studies [8,9], although certainly lower compared to neuropathological data [7].

The presence of CLs is known to be associated with neurological disability [15,16] and with cognitive impairment [15,18]. Indeed, our study showed a significant correlation between baseline CLs number and volume and cognitive dysfunction, with higher CLs count and volume being associated with lower SDMT scores. Total CLs count and volume at baseline also showed a significant association with normalized TBV.

The efficacy of OCR in reducing the risk of relapse and the development of new WMLs is well established through both clinical trials and real-world studies [22,23]. However, its effectiveness on the development of new CLs is still under investigation. This study is indeed one of the few specifically assessing the impact of OCR on the formation of new CLs. Another study evaluating the effect of OCR and sphingosine-1-phosphate (S1P) modulators on new CLs formation did not report a statistically significant difference between the two cohorts [33]; in particular, 18% of patients treated with OCR developed new CLs after two years of treatment initiation [33]. We should note that the previously mentioned study used conventional sequences rather than AIDIR [33]. Nevertheless, the already cited works report a comparable, if not superior, ability of AIDIR to detect cortical lesions compared with standard sequences used for cortical assessment [29,30]. Moreover, although disease duration was similar (approximately 10 years), the populations differed in baseline characteristics, including a higher median EDSS and the inclusion of patients with MS phenotypes other than RR-MS, suggesting that our cohort had an overall lower (and plausibly also cortical) disease burden [33]. In our cohort of RR-MS patients treated with OCR, only one patient developed a single new CL, in a context of clinical and cognitive stability; notably, this patient did not develop new WMLs during follow-up. This patient remains on OCR therapy. The rate of accumulation of new CLs was markedly lower than that observed in patients receiving other DMTs [[24], [25], [26],^34^,^35^], although, also in this case, direct comparisons are limited by substantial heterogeneity in study populations, disease phenotypes and MRI acquisition protocols. In particular, we should acknowledge that in all the aforementioned studies, cortical lesions were assessed using conventional DIR, FLAIR or MP2RAGE sequences.

In our study, 3 patients presented a clinical relapse and 14 patients experienced accrual of WMLs, which, however, were confined to the first months after initiation of OCR (mean 69 and 171 days respectively), mainly in patients with active disease prior to starting treatment, most of whom were transitioning from natalizumab or S1P modulators. This finding is consistent with existing evidence, which, while agreeing on the long-term efficacy of OCR in preventing the formation of new WMLs, also recognizes that previous DMT exposure, particularly to sequestering agents, was independently associated with an increased risk of MRI activity [23,36].

Thus, OCR confirms its role as a highly effective treatment in preventing the development of both WMLs and CLs, although, based on our study data, the kinetics of action appear to differ with respect to the development of these two lesion types. This difference in kinetics seems to support the existence of distinct pathogenetic mechanisms underlying the development of these two lesion types. This interpretation is further corroborated by neuropathological studies, which have shown that CLs are characterized by a relative lack of parenchymal lymphocyte infiltration, microglial activation, deposition of antibodies and complement proteins and blood–brain barrier disruption compared with WMLs [[37], [38], [39]].

Cortical pathology has also been studied in the form of cortical atrophy, a well-known surrogate marker of neurodegeneration. OCR is known to limit cortical atrophy compared to other DMTs [33,40]. In particular, in our study, which focused on the analysis of cortical volume and thickness in regions containing CLs, we excluded the development of lesion-driven cortical atrophy. To our knowledge, this is the first study to investigate the role of CLs in the development of locoregional cortical atrophy and to explore the efficacy of OCR in preventing its formation. Another example about the contribution of compartmentalized inflammation on the development of locoregional cortical atrophy is provided by a study on leptomeningeal inflammation; however, in this case, no mention was made of the role of DMTs [41].

Finally, in this study we outlined the utility of AIDIR sequences based on high-resolution 3D-FLAIR and T1-MPRAGE. The images obtained with this approach are reliable when compared with their original MRI-acquired counterparts, while allowing a reduction in acquisition time [29,30]. A broader availability of this technique would enable the implementation of additional acquisitions, providing a further tool useful both for differential diagnosis and for the prognostic stratification of MS patients.

This study has some limitations. First, it is a single-center cohort without a control group, including both treatment-naïve and previously treated patients with varying disease duration, which may limit the generalizability of the findings and make it difficult to isolate the effect of ocrelizumab. Second, the follow-up period of two years, while sufficient to capture short-term changes, may be too short to fully evaluate the long-term OCR effects on cortical pathology. Third, imaging techniques especially using 3T brain MRI, although reliable, may still fail to detect all CLs, particularly very small or subpial lesions; for this reason, we did not perform analyses aimed at distinguishing the separate contributions of leukocortical and non leukocortical lesions, however, we attempted to exclude patients presenting leukocortical lesions in the locoregional atrophy analysis. Fourth, in our study, only AIDIR sequences were available and conventional DIR sequences were not acquired; however they have shown their reliability in many studies [29,30]. Further studies may investigate the reproducibility in longitudinal settings. Nevertheless, the study cohort was homogeneous, all evaluations were performed using standardized protocols and the study is highly valuable for investigating longitudinal changes in a real-world cohort initiating ocrelizumab in routine clinical practice.

In conclusion, in a cohort of RR-MS patients treated with OCR, we demonstrated a strong effect of OCR on CLs formation and an expected efficacy on WMLs accrual, supporting the idea that CLs and WMLs development may be driven by distinct pathological mechanisms. OCR was associated not only with a reduced formation of new CLs over the two-year follow-up, but also with a potential attenuation of their impact, as suggested by a reduction in lesion-driven cortical atrophy. AIDIR sequences may represent a precious option to study the focal damage within cortical gray matter in clinical practice.

## Author contributions

NC: investigation, methodology, writing, data curation, visualization, formal analysis, data curation. GB: supervision, investigation, methodology, conceptualization, software, formal analysis, validation. EC: methodology, conceptualization, software, validation, data curation. VDB: investigation, data curation. SAQ: data curation. FB: data curation. EL: investigation, data curation. LN: investigation, data curation. SM: methodology, supervision. MC: supervision, investigation, methodology, conceptualization, software, methodology. MI: supervision, methodology, conceptualization, validation. CL: supervision, investigation, methodology, conceptualization, software, formal analysis, validation.

## Disclosures

GB received personal compensations from Novartis, Sanofi Genzyme, Roche, BMS and Merck. CL received travel grants from Roche, Merck, Sanofi and honoraria for speaking from Novartis, Roche, Merck, Horizon and BMS. SM is an employee of and shareholder in Roche. MC received personal compensations from Novartis, Sanofi Genzyme, Teva and consulting fees from Zambon. MI received grants NIH, NMSS, FISM; received fees for consultation from BMS; Janssen, Roche, Genzyme, Merck, Biogen and Novartis. CL received travel grants from Roche, Merck, Sanofi and honoraria for speaking from Novartis, Roche, Merck, Horizon and BMS. NC, EC, VDB, SAQ, FB, EL and LN have nothing to disclose.

## Declaration of competing interest

The authors declare the following financial interests/personal relationships which may be considered as potential competing interests:

Matilde Inglese reports financial support was provided by F. Hoffmann-La Roche Ltd. Giacomo Boffa reports a relationship with Novartis that includes: consulting or advisory. Giacomo Boffa reports a relationship with Sanofi that includes: consulting or advisory. Giacomo Boffa reports a relationship with F. Hoffmann-La Roche Ltd that includes: consulting or advisory. Giacomo Boffa reports a relationship with Bristol-Myers Squibb Company that includes: consulting or advisory. Giacomo Boffa reports a relationship with Merck KGaA that includes: consulting or advisory. Stefano Magon reports a relationship with F. Hoffmann-La Roche Ltd that includes: employment and equity or stocks. Maria Cellerino reports a relationship with Novartis that includes: consulting or advisory. Maria Cellerino reports a relationship with Sanofi that includes: consulting or advisory. Maria Cellerino reports a relationship with Teva Pharmaceuticals Industries Inc that includes: consulting or advisory. Maria Cellerino reports a relationship with Zambon SpA that includes: consulting or advisory. Matilde Inglese reports a relationship with National Institutes of Health that includes: funding grants. Matilde Inglese reports a relationship with NMSS that includes: funding grants. Matilde Inglese reports a relationship with Italian Multiple Sclerosis Foundation that includes: funding grants. Matilde Inglese reports a relationship with Bristol-Myers Squibb Company that includes: consulting or advisory. Matilde Inglese reports a relationship with Janssen Pharmaceuticals Inc that includes: consulting or advisory. Matilde Inglese reports a relationship with F. Hoffmann-La Roche Ltd that includes: consulting or advisory. Matilde Inglese reports a relationship with Sanofi that includes: consulting or advisory. Matilde Inglese reports a relationship with Merck KGaA that includes: consulting or advisory. Matilde Inglese reports a relationship with Biogen Inc that includes: consulting or advisory. Matilde Inglese reports a relationship with Novartis that includes: consulting or advisory. Caterina Lapucci reports a relationship with F. Hoffmann-La Roche Ltd that includes: speaking and lecture fees and travel reimbursement. Caterina Lapucci reports a relationship with Merck KGaA that includes: speaking and lecture fees and travel reimbursement. Caterina Lapucci reports a relationship with Sanofi that includes: travel reimbursement. Caterina Lapucci reports a relationship with Novartis that includes: speaking and lecture fees. Caterina Lapucci reports a relationship with Horizon that includes: speaking and lecture fees. Caterina Lapucci reports a relationship with Bristol-Myers Squibb Company that includes: speaking and lecture fees. If there are other authors, they declare that they have no known competing financial interests or personal relationships that could have appeared to influence the work reported in this paper.
